# Fully automated, deep learning, cardiac CT-based multimodal network for cardiovascular risk stratification in high-risk perioperative patients

**DOI:** 10.1093/ehjdh/ztag037

**Published:** 2026-03-04

**Authors:** Juan Lu, Gavin Huangfu, Abdul Ihdayhid, Mohammed Bennamoun, John Konstantopoulos, Simon Kwok, Kai Niu, Yanbin Liu, Gemma A Figtree, Matthew T V Chan, Craig R Butler, Vikas Tandon, Peter Nagele, Pamela K Woodard, Marko Mrkobrada, Wojciech Szczeklik, Yang Faridah Abdul Aziz, Bruce M Biccard, Philip James Devereaux, Tej Sheth, Michelle C Williams, David E Newby, Benjamin J W Chow, Girish Dwivedi

**Affiliations:** Medical School, The University of Western Australia, Perth, Australia; Harry Perkins Institute of Medical Research, Perth, Australia; Department of Computer Science and Software Engineering, The University of Western Australia, Perth, Australia; Medical School, The University of Western Australia, Perth, Australia; Harry Perkins Institute of Medical Research, Perth, Australia; Department of Cardiology, Fiona Stanley Hospital, Perth, Australia; Department of Cardiology, Fiona Stanley Hospital, Perth, Australia; Department of Computer Science and Software Engineering, The University of Western Australia, Perth, Australia; Artrya Ltd, Perth, Australia; Artrya Ltd, Perth, Australia; Department of Computer Science and Software Engineering, The University of Western Australia, Perth, Australia; Artrya Ltd, Perth, Australia; School of Engineering, Computer and Mathematical Sciences, Auckland University of Technology, Auckland, New Zealand; Department of Cardiology, Kolling Institute and Charles Perkins Centre, University of Sydney, Sydney, Australia; Department of Cardiology, Royal North Shore Hospital, Sydney, Australia; Department of Anesthesia and Intensive Care, The Chinese University of Hong Kong, Hong Kong SAR, China; Division of Cardiology, University of Alberta Hospital, Edmonton, Alberta, Canada; Department of Medicine, McMaster University, Hamilton, Canada; Department of Anaesthesia & Critical Care, The University of Chicago, Chicago, USA; Department of Radiology, Washington University School of Medicine, St. Louis, MO 63110, USA; Department of Medicine, Schulich School of Medicine & Dentistry, Western University, London, Ontario, Canada; Department of Intensive Care and Perioperative Medicine, Jagiellonian University Medical College, Krakόw, Poland; Departments of Radiology and Medicine, Faculty of Medicine, University of Malaya, Kuala Lumpur, Malaysia; Department of Anaesthesia and Perioperative Medicine, Groote Schuur Hospital, Faculty of Health Sciences, University of Cape Town, Cape Town, South Africa; Department of Medicine, McMaster University, Hamilton, Canada; Department of Medicine, McMaster University, Hamilton, Canada; Department of Medicine, University of Edinburgh, Edinburgh, UK; Department of Medicine, University of Edinburgh, Edinburgh, UK; Department of Medicine (Cardiology), University of Ottawa Heart Institute, Ottawa, Canada; Medical School, The University of Western Australia, Perth, Australia; Harry Perkins Institute of Medical Research, Perth, Australia; Department of Cardiology, Fiona Stanley Hospital, Perth, Australia; Department of Medicine (Cardiology), University of Ottawa Heart Institute, Ottawa, Canada; Department of Cardiology, Victor Chang Cardiac Research Institute, Crawley, WA 6009, Australia

**Keywords:** Convolutional neural networks, Coronary computed tomography angiography, Deep learning, Multimodal risk score, Perioperative risk

## Abstract

**Aims:**

Major adverse cardiac events (MACE) significantly impact perioperative morbidity and mortality. We aimed to develop a fully automated multimodal deep learning (DL) system integrating patient demographics, comorbidities, and coronary computed tomography angiography (CCTA) findings to optimize risk prediction.

**Methods and results:**

We included 639 patients undergoing CCTA as part of perioperative risk assessment for elective non-cardiac surgery. Convolutional neural networks automatically identified coronary artery disease reporting and data system (CAD-RADS) scores and segmented the left ventricle, aorta, and heart. These imaging features were combined with patient demographics and comorbidities to predict MACE risk. We evaluated the performance of our multimodal model against the revised cardiac risk index (RCRI) using gradient boosting decision tree modelling and area under the receiver operating characteristic (AUROC) curves. Among 639 patients (mean age 70 ± 9 years, 56% males, median RCRI 1), 61% underwent orthopaedic surgery, 27% vascular surgery and the rest abdominal/pelvic or spine surgery. 45 patients experienced MACE within 30 days. Automated CAD-RADS (AUROC = 0.69) demonstrated comparable performance to human analysis (AUROC = 0.67, *P* = 0.77). The multimodal DL system (AUROC = 0.82) outperformed CAD-RADS (delta-AUROC = 0.13, CI: 0.02, 0.26, *P* = 0.02), and RCRI (delta-AUROC =0.22, CI: 0.05, 0.46; *P* = 0.001) in predicting MACE and demonstrated robust sensitivity (83%) and specificity (79%).

**Conclusion:**

Our multimodal system built using automated CAD-RADS, anatomical segmentations and patient demographics outperforms both human expert and automated CAD-RADS for MACE prediction. This approach has the potential to enhance patient outcomes by leveraging the synergy between automated imaging and clinical data.

## Graphical Abstract

**Graphical Abstract:**
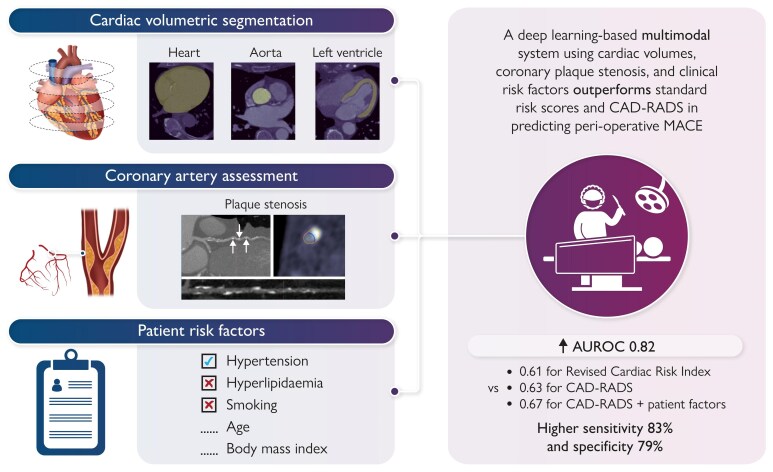

## Introduction

Cardiovascular (CV) disease remains the leading cause of death globally and is poised to rise further as we face increasing rates of obesity and the aging population.^1^ Clinical risk assessment tools such as the Framingham Risk Score and European Systemic Coronary Risk Evaluation rely on traditional risk factors to predict major adverse cardiovascular events (MACE) and serve a well-established role in primary prevention.^2,3^ However, systematic reviews and meta-analyses have shown there is a substantial residual risk that remains uncaptured by population-level scoring.^4,5^ The Revised Cardiac Risk Index (RCRI), a widely adopted tool for preoperative cardiac risk assessment, exemplifies these efforts.^6^ While the RCRI has demonstrated value in predicting perioperative MACE, studies have shown that it may underestimate risk in certain patient subgroups and specific surgical procedures.^7^ This limitation has prompted the need for more nuanced, patient-specific approaches to risk assessment that can capture the complex interplay of individual factors beyond those included in standardized scoring systems.

Coronary computed tomography angiography (CCTA) provides detailed non-invasive assessment of coronary plaque and stenoses, and has observed a sharp uptake in clinical practice, following its recommendation as a front-line diagnostic imaging test for coronary artery disease (CAD) in the latest guidelines.^8^ The Coronary Artery Disease Reporting and Data System (CAD-RADS) is a standardized method for reporting coronary stenoses on CCTA that has demonstrated robust prediction of MACE.^9^ It is currently assessed manually by radiologists using axial or reconstructed views and relies on visual estimation of luminal stenosis and identification of high-risk plaque.^10^

Advancements in the use of artificial intelligence (AI) and deep learning (DL) algorithms in the field of cardiac imaging over the last decade have expanded the possibilities for streamlining CV risk assessment. AI can better handle complex data with large numbers of inputs, and thus is capable of processing and integrating diverse data modalities, including radiographic images, tabular clinical records, and 3D volumetric measurements of anatomical structures, akin to that of human intelligence.^11^ Ultimately, multimodal DL techniques provide an opportunity to improve and personalize CV risk prediction.

We aimed to evaluate the prognostic value of a fully automated multimodal risk network that integrates automated CAD-RADS, patient clinical and volumetric data in predicting short-term perioperative MACE. We hypothesize that our novel model will surpass the current standard of care, including clinical risk scores such as the RCRI, which is used for perioperative risk prediction.^6^

## Methods

### Study population

The study cohort consisted of 639 patients who underwent CCTA as part of perioperative risk assessment for elective non-cardiac surgery, enrolled in the international, prospective, multicentre study named coronary computed tomography angiography vascular events in non-cardiac surgery patient’s cohort evaluation (coronary CTA VISION). All patients provided informed consent, and the study was approved by each participating centre’s ethics committees. The clinical trial number is NCT00512109. The VISION cohort included patients who were undergoing vascular, orthopaedic, abdominal or pelvic, major spine, and other types of non-cardiac surgeries. Details of study design, methodology, clinical variables, and CCTA protocol have been published elsewhere.^10^ All patients had a history of, or were at risk of, atherosclerotic disease.

**Figure 1:**
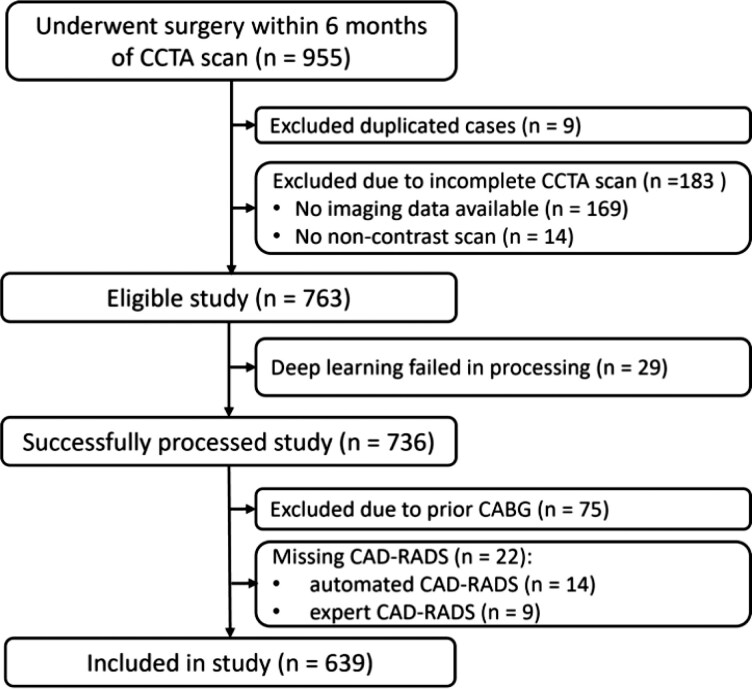
Schema showing selection of the study population. CCTA, coronary computed tomography angiography; CAD-RADS, coronary artery disease—reporting and data system.

As seen in *Figure 1*, patients were excluded if they had incomplete scans, unsuccessful DL processing, no expert-based CAD-RADS analysis or history of coronary artery bypass grafting (as the automated CAD-RADS model has not been trained for assessing grafts). CCTA reporting was performed by physicians blinded to the clinical data, and treating physicians did not have access to CCTA findings until 30 days after surgery, except for cases with left main stenosis ≥50% which were reported immediately and excluded from the study. The primary outcome was 30-day MACE, defined as a composite of CV death and non-fatal myocardial infarction (MI) within 30 days after surgery. MI diagnosis was defined according to the third universal definition criteria.^12^

### Automated CAD-RADS

A DL algorithm was implemented, consisting of a three-dimensional (3D) convolutional neural network (CNN) that utilizes a U-Net architecture. The process of detecting coronary vessels, extracting centrelines, and segmenting vessel walls and lumens was automated using unsupervised DL algorithms.^13^ Images underwent voxel-level segmentation with the contouring of the vessel wall and lumen being successfully achieved. The segmentation of lumen was carried out using the straightened multiplanar reformation of the tracked coronary artery. This involved extracting coronary centrelines by identifying potential seed points within the coronary artery, followed by tracking these points and connecting them to form a comprehensive coronary tree. A classifier was then employed to label the extracted arteries based on a range of features. Subsequently, three 3D CNNs are used to classify the severity of coronary artery stenosis. Discrepancies between the lumen and the vessel wall were identified as regions of plaque, and stenosis was determined by comparing these areas with a healthy reference section. The comparison yielded a maximal coronary stenosis measurement, expressed as a percentage of vessel diameter indicating the extent of narrowing. Stenosis severity was assessed for each coronary segment based on the maximal diameter stenosis and categorized according to CAD-RADS defined as: 0 (0%), 1 (1–24%), 2 (25–49%), 3 (50–69%), 4 (70–99%), and 5 (100%).^9^

**Figure 2:**
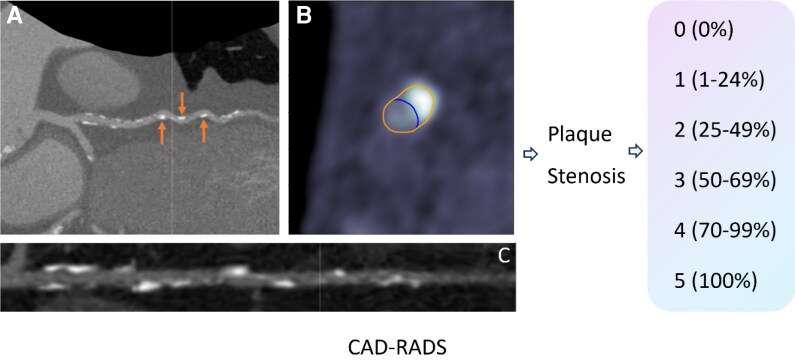
Panel A displays a lesion located in the mid-segments of the LAD. Panel B presents a cross-sectional view where plaque characterization and contouring have been performed automatically by the deep learning CAD-RADS system. Panel C showed a long axis view of the lesion location. CAD-RADS, coronary artery disease—reporting and data system.

*Figure 2* presents a representative example demonstrating how the automated CAD-RADS system effectively categorizes stenosis from various viewpoints. The varied views have shown that the automated CAD-RADS system performed well in categorizing stenosis. Categorical CAD-RADS agreement between expert readers and DL was 43.8%.

### Training, validation, and testing

We randomly allocated 80% of the data in coronary CTA VISION study to the training set and the remaining 20% to the testing set. At the training phase, the training set was further split into training (90%) and internal validation (10%) sets.

### Segmentation of cardiac structures

**Figure 3:**
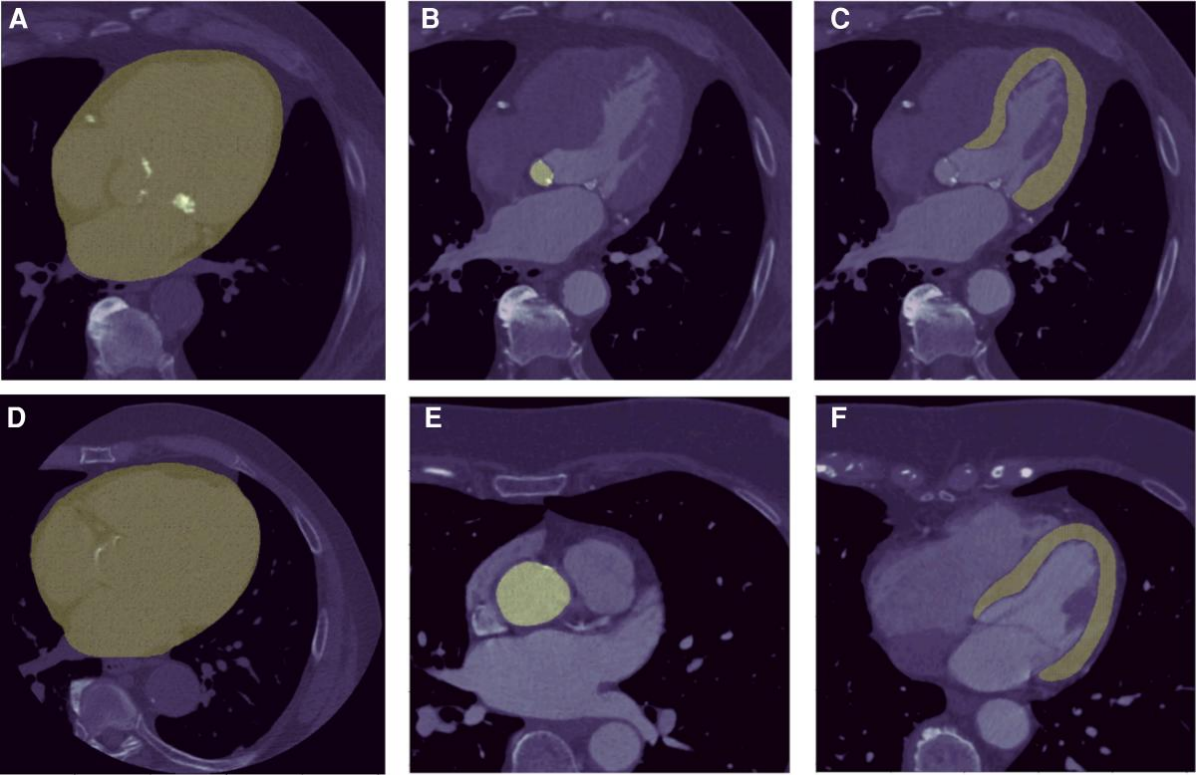
Example cases of heart, aorta and LV segmentation. Panels (*A*) and (*D*) demonstrate successful segmentation of the heart area in non-contrast scans. Panels (*B*) and (*E*) illustrate the aorta while panels (*C*) and (*F*) show the segmentation of the LV area in systole and diastole, respectively. LV: left ventricle.

*Figure 3* presents example cases of cardiac structure segmentation, illustrating our methodology of delineating the heart, aorta, and left ventricle (LV) in CCTA scans (Panels A and D). This comprehensive framework integrates data from multiple sources, i.e. segmentation masks derived from both contrast-enhanced and non-contrast CT scans of heart, LV and aorta, along with tabular data including patient demographics, clinical information, and comorbidity history. In the initial stage, heart, aorta, and LV segmentations are created based on 2D masks generated by separate 2D CNN model. Specifically, the segmentation of the heart is generated from non-contrast CT scans, and the segmentations for aorta and LV are derived from contrast-enhanced CT scans. These segmentation masks are applied to the original DICOM slices, each with a resolution of 512 × 512 pixels. The numbers of slices vary among individual cases reflecting the expected natural variation of these structures between individuals. The example number of slices and shape of segmentation presented in *Figure 3* are only for illustrative purposes. Subsequently, the processed embeddings from each modality serve as inputs to the Perceiver Encoder (see Supplementary material online, *Figure S1*). The output latent vectors from the encoder are then concatenated and input into the Perceiver Decoder, which is responsible for predicting the probability of MACE risk.^14^

### Tabular input

We included a broad spectrum of variables important to CV risk assessment, such as patient demographics, history of vascular disease, vascular risk factors, patients with 3 or more vascular risk factors, and history of stress nuclear imaging or stress echocardiography in the last 6 months. Body mass index (BMI) was the only feature that contains missing values, with 8.6% data missing. We employed Multiple Imputation by Chained Equations (MICE)^15^ method on the input data to impute missing values. The detailed list of input variables is provided in Supplementary material online, *Table S1*.

### Multimodal deep learning

**Figure 4:**
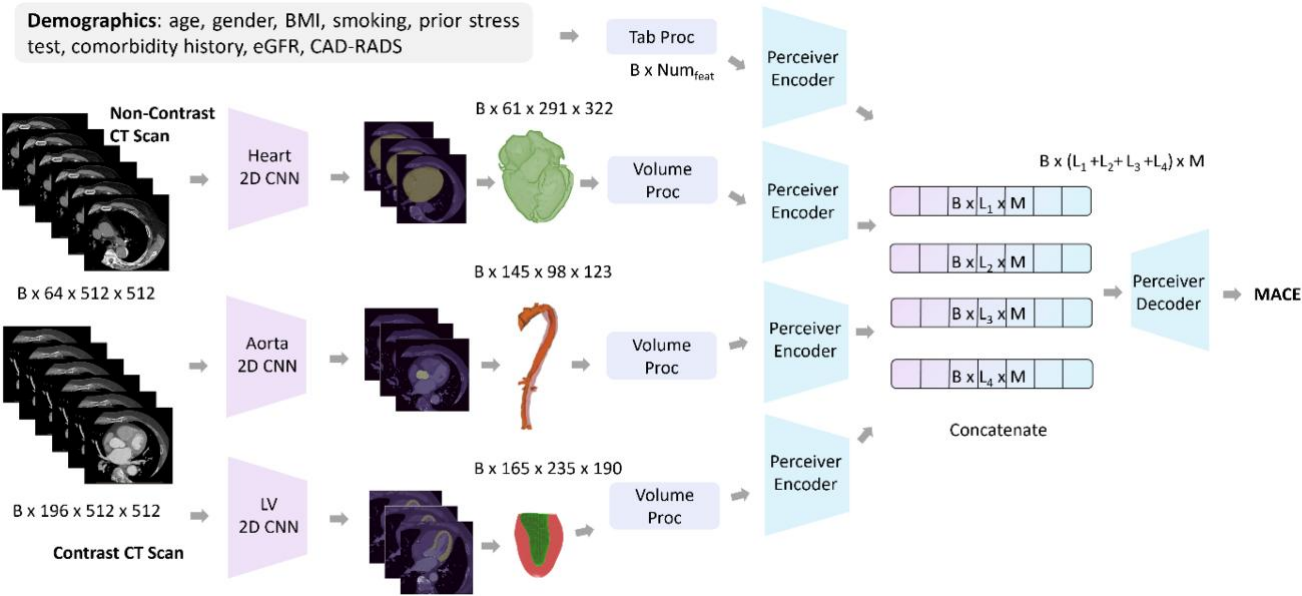
Illustration of the proposed multimodal deep learning system for MACE risk prediction. BMI, body mass index; eGFR, estimated glomerular filtration rate; CAD-RADS, coronary artery disease—reporting and data system; CT, computed tomography; 2D, two dimensional; CNN, convolutional neural networks; LV, left ventricle; MACE, major adverse cardiovascular events.

*Figure 4* showed the proposed multimodal framework for predicting the patient risks of MACE. Our framework uniquely combines diverse data sources and modalities: segmentation masks from both contrast and non-contrast CCTA scans, tabular data including patient demographics and clinical history. Detailed modality processing is listed in the Supplementary materials. The 2D CNN model was employed in segmenting different cardiac structures, which are key anatomical features for patient risk assessment. Details of perceiver encoder, perceiver decoder and hyperparameter tuning are included in the Supplementary Methods.

### Statistical analysis

Continuous variables were summarized as mean ± standard deviation (SD) or median [Q1–Q3] depending on normality, while categorical variables were presented as frequencies with percentages. Continuous variables were compared using the Student’s *t*-test for normally distributed variables or the Wilcoxon rank-sum test for non-normally distributed variables. The categorical variables are compared using chi-square testing. We utilized the gradient boosting decision tree (GBDT) model^16^ to compare the performances of different risk scores, automated and expert-interpreted CAD-RADS for MACE within 30 days. The predictive and incremental value of these scores when added to patient demographic and comorbidity history were compared using area under receiver operating characteristic (AUROC) curve analysis. Comparisons between models were performed using DeLong’s test for paired AUROC curves. Additionally, sensitivity, specificity and negative predictive value were calculated for each model configuration. These metrics are particularly relevant to identifying patients who are unlikely to experience MACE. Confidence intervals for model performance were derived using a non-parametric bootstrap based on 1000 resamples of the testing set. Univariate Cox regression analyses were conducted to evaluate the prognostic significance of expert and automated CAD-RADS in predicting MACE. Calibration was assessed by the calibration plot (observed vs. predicted risk). Clinical utility was evaluated using decision curve analysis to estimate net benefit, with 7% (for patients with 2 RCRI predictors) as the primary clinically relevant threshold.^17,18^ We employed permutation feature importance to assess the contribution of individual features to the overall predictive accuracy for MACE. For a sensitivity analyses, we repeated the prediction model analyses using two alternative outcome definitions: (i) an expanded 30-day composite endpoint including MACE and heart failure (HF) hospitalization, and (ii) type 2 MI alone (defined as non-fatal MI that did not result in revascularization or CV death). All statistical analyses were undertaken using Python Version 3.9 and SAS Version 9.4 software. Statistical significance was defined as *P*-value < 0.05.

## Results

### Study population

Baseline characteristics of the study population, stratified by MACE status, are shown in *Table 1*. Over 30-day of follow up, 45 (7.0%) patients experienced MACE. These patients were older, had lower BMI and were more likely to have 3 or more CV risk factors (22% vs. 47%, *P* = 0.0007). The mean age was 70 ± 9 years and 56% were male. Nearly, one fourth of patients had a history of CAD and one third had peripheral vascular disease. Around 47% of patients had 3 or more risk factors, with the most prevalent being hypertension (89%), hypercholesterolaemia (79%) and diabetes (40%). Most patients were on CV medications, including 73% statins and 61% angiotensin-converting enzyme inhibitors (ACEi). Orthopaedic surgery (61%) was the most common elective surgery, followed by vascular surgery (27%) then abdominal/pelvic surgery (9%). Three-quarters of patients had an RCRI of 0 or 1, with only five patients in the highest risk group of 4.

**Table 1:** Characteristics of study population

	Study population (*n* = 639)	MACE (*n* = 45)	Event free survivors (*n* = 594)	*P*-value
**Demographics**				
Age (mean ± SD)	69.9 ± 8.7	72.5 ± 6.5	69.7 ± 8.8	< 0.001
Male	361 (57)	38 (64)	398 (59)	0.40
BMI (median Q1—Q3)	28.7 [25.1, 33.2]	28.4 [25.8,32.4]	28.7 [25.1, 33.3]	< 0.001
**Medical history**				
Peripheral vascular disease	199 (31)	23 (51)	176 (30)	0.003
Coronary artery disease	157 (25)	17 (38)	140 (24)	0.033
Congestive heart failure	20 (3)	4 (9)	16 (3)	0.021
Diabetes	258 (40)	24 (53)	234 (39)	0.06
Smoker	167 (26)	8 (18)	159 (27)	0.12
Hypertension	566 (89)	42 (93)	524 (88)	0.29
Atrial fibrillation	24 (4)	2 (4)	22 (4)	0.80
COPD	54 (9)	7 (16)	47 (8)	0.07
TIA	62 (10)	5 (11)	57 (10)	0.74
Stroke	65 (10)	5 (11)	60 (10)	0.83
Hypercholesterolemia	506 (79)	34 (76)	472 (80)	0.53
Prior PCI	11 (2)	3 (7)	8 (1)	0.008
**Pre-operation eGFR**				0.07
<30 or dialysis at baseline	2 (0)	0 (0)	2 (0)	
30–45	33 (5)	6 (13)	27 (5)	
45–60	98 (15)	5 (11)	93 (16)	
≥60	501 (78)	34 (76)	467 (79)	
≥3 risk factors^a^	298 (47)	10 (22)	288 (49)	<0.001
**Medications**				
Aspirin	263 (41)	27 (60)	236 (39)	0.011
ACE inhibitor	387 (61)	29 (64)	358 (60)	0.58
Beta blocker	252 (39)	28 (62)	224 (38)	0.001
Statin	467 (73)	37 (82)	430 (72)	0.15
Oral anticoagulation	16 (3)	2 (4)	14 (2)	0.39
**Surgical procedures**				
Vascular	170 (27)	18 (40)	152 (26)	0.05
Orthopedic	387 (61)	24 (53)	363 (61)	0.30
Abdominal/pelvic	57 (9)	4 (9)	53 (9)	0.99
Major spine	7 (1)	0 (0)	7 (1)	0.46
Other	30 (5)	0 (0)	30 (5)	0.12
**RCRI**				<0.001
0	249 (39)	12 (27)	237 (40)	
1	246 (39)	17 (38)	229 (39)	
2	115 (18)	11 (24)	104 (18)	
3	24 (4)	5 (11)	19 (3)	
4	5 (1)	0 (0)	5 (1)	

^a^Patients qualified based on risk factors alone, that is ≥3 of 6 risk factors including history of diabetes, age ≥70, history of smoking within 2 years of surgery, history of treatment for hypercholesterolemia, history of TIA, and history of hypertension.

MACE, major adverse cardiovascular events; SD, standard deviation; BMI, body mass index; COPD, chronic obstructive pulmonary disease; TIA, transient ischaemic attack; PCI, percutaneous coronary intervention; eGFR, estimated glomerular filtration rate; ACE, angiotensin-converting enzyme; RCRI, revised cardiac risk index.

### Performance of automated CAD-RADS

CCTA findings, classified by expert, revealed that 71 (11%) patients had an absence of CAD (CAD-RADS 0), 293 patients had minimal to mild non-obstructive CAD (CAD-RADS 1–2), 87 (14%) patients had moderate stenosis (CAD-RADS 3), 127 (20%) patients had severe stenosis (CAD-RADS 4), and 61 (10%) patients had total coronary occlusion (CAD-RADS 5). The correlation between human expert and DL based CAD-RADS can be seen in Supplementary material online, *Table S2*.

**Figure 5:**
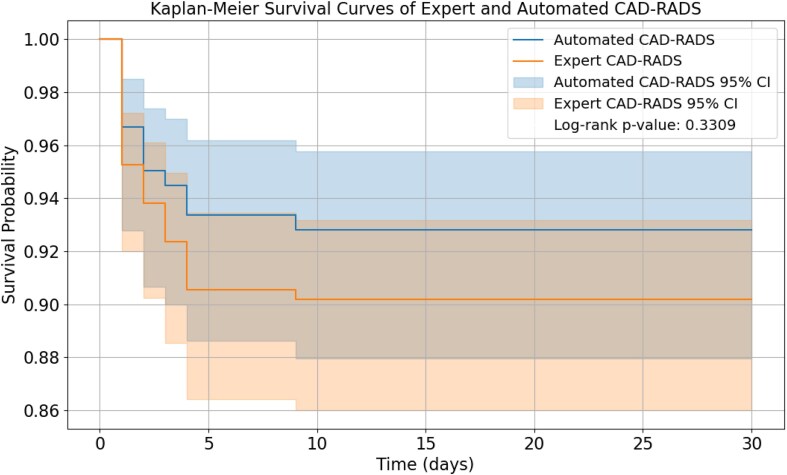
Kaplan–Meier survival curves of expert and automated CAD-RADS. CAD-RADS, coronary artery disease—reporting and data system.

*Table 2* presents the comparison of automated CAD-RADS, expert CAD-RADS, the RCRI, and multimodal DL systems in predicting MACE using AUROC with sensitivities, specificities, and NPV based on Youden’s index. Patient data and RCRI alone had an AUROC of 0.62 and 0.61, respectively, for predicting MACE, and this was similar to both expert and automated CAD-RADS (*P* = 0.90). Expert and automated CAD-RADS yielded similar AUROCs (0.61 vs. 0.63, *P* = 0.73). Univariate Cox regression revealed a hazard ratio of 1.30 (95% CI: 1.08–1.60) for expert CAD-RADS (*P* = 0.01) predicting MACE. Similarly, the hazard ratio for automated CAD-RADS was found to be 1.28 (*P* = 0.02). Kaplan–Meier curves of expert and automated CAD-RADS are presented in *Figure 5*.

**Table 2:** Performance of different models in predicting 30-day MACE

Input	AUC-ROC ↑	Sensitivity^a^↑	Specificity^a^↑	NPV^a^↑	F1-score^a^↑
**CAD-RADS vs. RCRI and patient data (demographics, history of vascular disease, and vascular risk factors)**					
Patient data	0.62 (0.43, 0.79)	0.89 (0.57, 0.98)	0.36 (0.28, 0.45)	0.98 (0.89, 0.99)	0.17
RCRI only	0.61 (0.44, 0.77)	0.78 (0.45, 0.94)	0.48 (0.39, 0.57)	0.97 (0.89, 0.99)	0.18
CAD-RADS only (expert)	0.61 (0.43, 0.79)	1.0 (0.70, 1.00)	0.15 (0.10, 0.23)	1.0 (0.82, 1.00)	0.15
CAD-RADS only (automated)	0.63 (0.43, 0.82)	0.89 (0.57, 0.98)	0.30 (0.23, 0.39)	0.97 (0.86, 0.99)	0.16
RCRI + patient data	0.66 (0.47, 0.85)	0.56 (0.27, 0.81)	0.71 (0.63, 0.79)	0.96 (0.89, 0.98)	0.21
CAD-RADS (expert) + patient data	0.67 (0.50, 0.84)	0.56 (0.27, 0.81)	0.78 (0.68, 0.83)	0.96 (0.90, 0.98)	0.24
CAD-RADS (automated)) + patient data	0.69 (0.52, 0.86)	0.89 (0.57, 0.98)	0.45 (0.37, 0.54)	0.98 (0.91, 0.99)	0.20
**Multimodal**					
Tabular data [CAD-RADS (DL) + patient data]	0.69 (0.52, 0.87)	0.78 (0.45, 0.94)	0.51 (0.38, 0.56)	0.97 (0.89, 0.99)	0.18
LV + Heart + Tabular	0.72 (0.57, 0.87)	0.89 (0.57, 0.98)	0.53 (0.45, 0.62)	0.99 (0.92, 0.99)	0.18
LV + Aorta + Tabular	0.76 (0.62, 0.90)	0.89 (0.57, 0.98)	0.58 (0.49, 0.66)	0.99 (0.93, 0.99)	0.23
LV + Heart + Aorta + Tabular	0.82 (0.63, 0.92)	0.83 (0.55, 0.94)	0.79 (0.69, 0.85)	0.98 (0.94, 0.99)	0.43

^a^Threshold were chosen based on the max Youden-index.

LV, left ventricle; CI, confidence interval; CAD-RADS, coronary artery disease—reporting and data system.

The numbers in parentheses indicate the 95% confidence intervals (CI).

Combining patient data with risk scores increased the AUROC, with the highest performance observed with automated CAD-RADS (AUROC 0.69; 95% CI: 0.52–0.86, sensitivity 89%, specificity 45%, NPV 98%), and the performance of expert CAD-RADS was similar (AUROC 0.67; 95% CI: 0.50–0.84, *P*-value = 0.78, sensitivity 56%, specificity 77%, NPV 96%). Thresholds used were listed in Supplementary material online, *Table S3*. The mean computation time was 110 ± 43 and 30 ± 4 s for stenosis detection per CCTA scan.

The multimodal system comprising patient data, automated CAD-RADS and segmentation of the three structures had an AUROC of 0.82, and this was significantly higher than the combination of patient data and automated CAD-RADS (delta AUROC = 0.13, CI: 0.02–0.26, *P* = 0.02). The model had a sensitivity of 83%, specificity of 79%, NPV of 98%, and demonstrated the highest F1 score of 0.43. The multimodal system also outperformed RCRI (delta AUROC = 0.22, CI: 0.05, 0.46; *P* = 0.001) in predicting MACE. It showed superior calibration and the greatest net benefit in decision curve analysis across clinically relevant thresholds compared with alternative models (see Supplementary material online, *Figures S2* and *S3*).

In both sensitivity analyses of type 2 MI alone and the composite endpoint including HF hospitalization, discrimination by the multimodal network was marginally higher than RCRI and CAD-RADS but did not reach significance (see Supplementary material online, *Tables S4* and *S5*). The relative ranking of models remained unchanged, with the multimodal network demonstrating the highest AUROC in predicting type 2 MI (0.75 vs. 0.61 for CAD-RADS and patient data), and composite of MACE and HF hospitalization (0.80 vs. 0.66 for CAD-RADS and patient data).

### Feature importance

**Figure 6:**
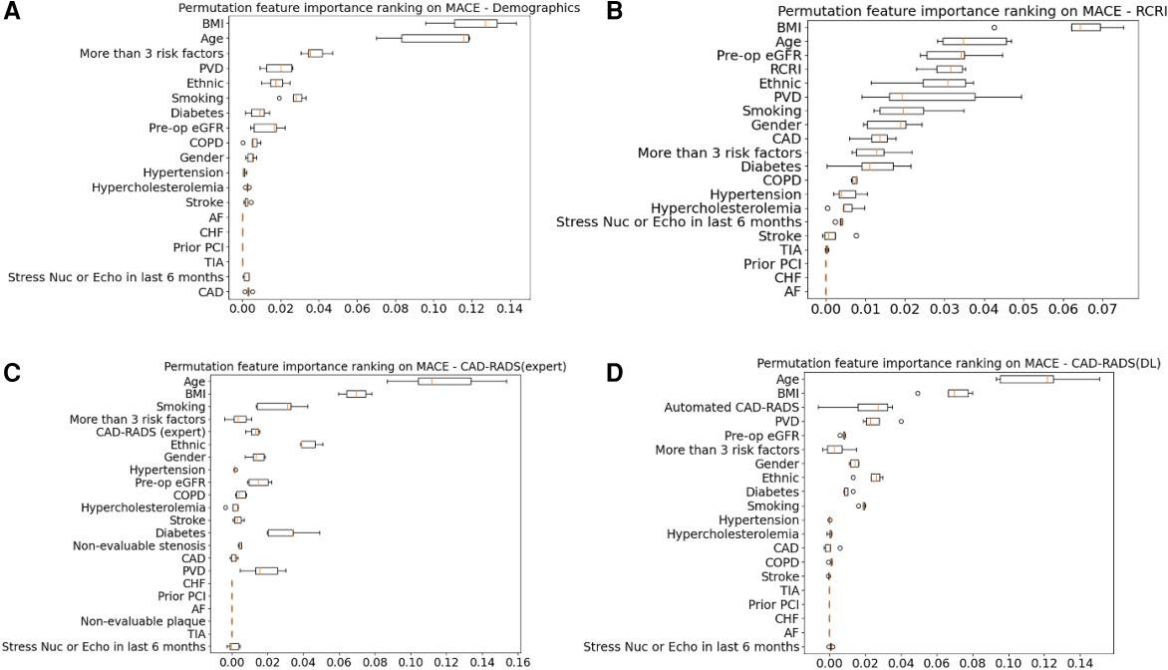
Permutation feature importance of RCRI and CAD-RADS. Panel A displays the feature importance based on patient demographics and comorbidity history. Panel B presents the feature importance when using RCRI and patient data as input to the model. Panel C demonstrates the feature importance with expert CAD-RADS and patient data as inputs. Panel D reveals the feature importance utilizing automated CAD-RADS and patient data as inputs. MACE, major adverse cardiovascular events; BMI, body mass index; PVD, peripheral vascular disease; eGFR, estimated glomerular filtration rate; COPD, chronic obstructive pulmonary disease; AF, atrial fibrillation; CHF, congestive heart failure; PCI, percutaneous coronary intervention; TIA, transient ischaemic attack; CAD, coronary artery disease; RCRI, revised cardiac risk index.

The feature importance ranking of RCRI, expert CAD-RADS, and automated CAD-RADS in predicting MACE are demonstrated in *Figure 6*. Although BMI and age were the most significant predictors of MACE, regardless of the change of input features, both the expert CAD-RADS and automated CAD-RADS are ranked within the top-5 most important features. Prior percutaneous coronary intervention, atrial fibrillation, and heart failure, were ranked low within the selected feature.

## Discussion

This is the first study to leverage a multicentre dataset and structural cardiac assessment to develop a fully automated DL, CCTA-based multimodal risk assessment network that is capable of robustly predicting short-term MACE and outperforms clinical perioperative risk scores. The main findings are that automated CAD-RADS predicts 30-day MACE comparable to expert analysis (*P* = 0.90), and its predictive capacity is significantly enhanced by the addition of cardiac structure segmentation as well as patient information using DL processes that can be performed in a fraction of the time (*P* = 0.023). These results highlight the potential utility and cost-effectiveness of AI-based multimodal frameworks in clinical practice to service an expanding patient load.

The use of CCTA in peri-operative risk assessment has traditionally observed high negative predictive values; however, a major downfall has been its poor specificity leading to gross overestimation of risk that may result in delay/cancellation of surgery and unnecessary additional testing.^17^ Compared with Koshy *et al*.’s meta-analysis of four CCTA studies demonstrating a pooled specificity 0.35 and AUROC 0.77,^19^ our multimodal tool delivered a notably higher AUROC and specificity while maintaining a high negative predictive value. These improvements stem from the ability of the DL software to seamlessly integrate multiple data forms to predict risk rather than reliance on singular modalities such as previous CCTA trials.

Several studies have demonstrated comparable performance of automated CAD-RADS to human experts for characterizing stenosis, however its translation into predicting patient outcomes remains limited.^20–22^ Lin *et al*. showed DL-based obstructive stenosis ≥50% was associated with an increased risk of MI, exhibiting an AUROC of 0.70 on sub-analysis.^23^ Unlike Lin *et al.*, our study examined the performance of the range of CAD-RADS scores rather than a binary classification to predict CV death as well as MI. The relatively low AUROC observed with individual inputs is likely due to the short follow up period of within 30 days of surgery and is consistent with other literature for perioperative risk with meta-analyses of RCRI showing pooled AUROCs of 0.62 for mortality and 0.75 for cardiac events.^7^

Segmentation data, particularly of the LV, demonstrated the greatest AUROC of all modalities tested. Studies in pre-operative echocardiography show LV systolic dysfunction and hypertrophy were strong, independent predictors of cardiac events, and their incorporation into risk models improved the detection of perioperative complications better than clinical variables.^24^ The findings of our study support this as LV segmentation on CCTA essentially provides a surrogate assessment of structural heart disease, with studies showing it is equivalent or may be more accurate than echocardiography.^25^ Unsurprisingly, we observed that the inclusion of segmentation data on our multimodal network contributed substantially to the high AUROC of 0.821. CCTA provides clinicians with abundant information of the coronary vasculature and pericoronary tissue.^26^ Beyond stenosis and cardiac structure segmentation, CCTA identifies plaque characteristics like high-risk features of spotty calcium, low attenuation, positive remodelling, and napkin ring sign, and is capable of measuring quantitative plaque volume.^26^ Alongside, pericoronary adipose tissue attenuation and epicardial adipose tissue, these biomarkers represent coronary inflammation and vulnerability that have emerged as robust predictors for future MACE, incremental to traditional obstructive disease and calcium score.^27,28^ Body mass index was an important predictor of MACE in our study, and has been shown to have a U-shaped relationship whereby extremes of body size confer greater risk.^29,30^ This is unsurprising as low BMI is a surrogate marker of frailty, and obese individuals have additional surgical and anaesthetic considerations. An important next step would be assessing the utility of these biomarkers in a multimodal network to predict MACE.

The integration of AI in CV imaging has advanced substantially over the past decade and parallels the growing need for improved workflow efficiency to match the rising demand. One of the major drawbacks of traditional DL algorithms has been its focus on one data modality limiting its ability to replicate human intelligence, which inherently perceives information from various data forms.^31^ Our study capitalizes on fusion modelling using convolutional neural networks and a latent array to bypass the need for manual extraction of clinical and imaging data, which is labour and time consuming,^32^ resulting in a fully automated model with superior discriminatory potential for perioperative CV risk assessment. This supplements work performed in various medical fields like oncology and COVID19 research whereby multimodal DL techniques have already been used effectively for risk stratification purposes.^33^ The benefits are multiple; first, by offloading the need for human analysis, the operator’s role can be re-assigned to other tasks thus making cardiac imaging more widely available.^34^ Secondly, our model can be readily applied to large populations for external validation and primary prevention screening where patient selection is broad. Finally, AI overcomes human error due to its high reproducibility and can act as an educational tool to train physicians in interpreting cardiac CT.^34^ Further studies are recommended exploring the potential performance enhancements from other querying processes, like embedding of tabular data, on MACE prediction.

### Limitations

The study leveraged a well characterized multicentre cohort where a large number of CCTA scans from several different vendors were used for training, validating, and testing of the multimodal network, reflecting real world practice. However, there are several limitations that must be acknowledged. The study focused on 30-day MACE in a perioperative setting resulting in a relatively small event rate, wide confidence intervals and limiting its generalizability. The predictivity of the novel model in other populations and for longer term outcomes should be examined in future studies. Other relevant CV outcomes such as arrhythmias and stroke were not available. Non-fatal MI was based on the Third Universal Definition. Features such as high-risk plaques, low attenuation plaque, and napkin-ring signs, which are critical biomarkers from CCTA influencing patients’ risk for MACE, were not included. Future studies should incorporate these features to enhance the model's predictive accuracy and clinical relevance. Although patients with advanced chronic kidney disease (creatinine clearance <35 mL/min) were excluded, contrast-enhanced CT confers risk of contrast-induced nephropathy and harms of ionizing radiation, thus a tailored patient-centred approach to risk and benefit assessment of peri-operative CCTA is required in clinical practice. While CAD-RADS was available as a reference standard, concordance between human and automated CAD-RADS was likely underestimated because the human reference set contained no CAD-RADS 0 cases and the automated system did not differentiate CAD-RADS 1 from 2, resulting in an inherent label-space mismatch. Segmentation of the LV, aorta and heart could not be cross-validated with findings from echocardiography or cardiac MRI. Scans with metal artefact and high levels of noise were excluded, as were patients with history of bypass grafting. Finally, given that the segmentation algorithm is proprietary, independent external validation is essential before clinical implementation can be recommended.

## Conclusions

A novel, fully automated multimodal network utilizing plaque stenosis and segmentation on cardiac CT and patient information provides rapid, robust prediction of short-term MACE in the perioperative setting for high-risk patients. It outperforms any single modality data, including the current standards of expert-based CAD-RADS and RCRI score. Future studies exploring its potential impact on workflow for cardiac CT stations, the downstream implications for clinical management in the perioperative setting, and its role in other populations are warranted.

## Supplementary Material

ztag037_Supplementary_Data

## Data sharing statement

Data generated or analysed during the study are available from the corresponding author by request.
