# Supplementary material for: Fully automated, deep learning, cardiac CT-based multimodal network for cardiovascular risk stratification in high-risk perioperative patients
Source: Eur Heart J Digit Health. 2026 Mar 4;7(3):ztag037. doi: 10.1093/ehjdh/ztag037 (PMC12980501; doi:10.1093/ehjdh/ztag037)

**SUPPLEMENTARY MATERIALS**

**SUPPLEMENTARY METHODS**

**Data processing**

*Tabular data*

Continuous features were initially encoded using piece-wise linear encoding. Subsequently, a trainable embedding ($ⅇ_{t}, t=1, \ldots, T$, for *T* bins) was applied for each bin. Finally, we aggregated the embeddings of each bin using weights ($\omega$) and biases (ν), as $E= \nu+ \sum_{t=1}^{T} e_{t}\cdot\omega_{t}$. Categorical features were fed into a parametric embedding, consisting of a feed-forward network with a learnable embedding layer. The embeddings from continuous and categorical features were flattened, concatenated, and inputted into the Perceiver encoder.

*Volume*

We standardized the voxel spacing across all CT segmentations to accommodate the constraints imposed by GPU memory limitations. Specifically, the voxel spacing for the heart was doubled in the coronal and sagittal planes, while for the aorta and LV, the spacing was doubled in all three dimensions. After normalizing the voxel values, three dimensional (3D) Fourier positional encoding was applied to effectively capture the spatial relationships within the data. The encoded data was then fed into the Perceiver encoder.

**Perceiver encoder**

The embeddings were first input into the cross-attention layer of the Perceiver encoder, where they underwent a cross-attention operation with the encoder’s latent array (Supplementary Figure S1). In this operation, the predefined learnable latent array produces queries (Q), while the processed input embeddings produce keys (K) and values (V). Following cross-attention, self-attention was applied on the latent features from the cross-attention layer. Subsequently, the encoder generates a latent vector, which is dimensionally equivalent to that of Q. This latent vector encapsulates the final hidden states derived from the latent features. This approach effectively eliminates the quadratic dependency on the size of the input data (volumetric or tabular), making our pipeline efficient and fast.

**Perceiver decoder**

The latent vectors of both tabular and volume was then concatenated to form a comprehensive representation. This unified vector was then fed into the Perceiver decoder, which implements a cross-attention layer to transform the concatenated latent vectors into classification logits. During this process, trainable embeddings are used to generate the Q, while the latent vectors are employed to produce K and V.

**Hyperparameter tuning**

We utilized Bayesian hyperparameter tuning^15^ and 5-fold cross-validation to optimize the performance of GBDT. We optimized for learning rate, number of leaves, feature fraction, bagging fraction, minimum data in leaf, max depth, max bin, minimum sum hessian in leaf, and subsample. We conducted grid search to identify the optimal hyperparameter for our multi-modal Perceiver model.

**Table S1. Detailed input variables.**

| Input variables | age, gender, BMI, stress nuclear or Echo test in last 6 months, CAD, PVD, COPD, stroke, congestive heart failure, diabetes, smoking, hypercholesterolemia, TIA, hypertension, ethnic, prior PCI, AF, pre-operation GFR, patients with 3 or more vascular risk factors |
| --- | --- |

**Table S2 4. Expert read vs. deep learning model on per-patient CAD-RADS**

| **Category** | | **Human expert CAD-RADS (n)** | | | | | | **Total** |
| --- | --- | --- | --- | --- | --- | --- | --- | --- |
|  |  | 0 | 1 | 2 | 3 | 4 | 5 |  |
| **automated CAD-RADS (n)** | 0 | 0 | 61 | 5 | 1 | 4 | 0 | 71 |
|  | 1, 2 | 0 | 99 | 95 | 58 | 40 | 1 | 293 |
|  | 3 | 0 | 14 | 23 | 28 | 22 | 0 | 87 |
|  | 4 | 0 | 1 | 15 | 53 | 58 | 0 | 127 |
|  | 5 | 0 | 2 | 7 | 19 | 33 | 0 | 61 |
| **Total** | | 0 | 177 | 145 | 159 | 157 | 1 | 639 |

CAD-RADS, coronary artery disease – reporting and data system

**Table S3. Threshold for different models in predicting MACE**

| **Input** | **Threshold** | **Sensitivity*↑** | **Specificity*↑** | **NPV*↑** | **F1-score*↑** |
| --- | --- | --- | --- | --- | --- |
| **CAD-RADS versus RCRI and patient data (demographics, history of vascular disease, and vascular risk factors)** | | | | | |
| **Patient data** | 0.25 | 0·89  (0·57, 0·98) | 0·36  (0·28, 0·45) | 0.98  (0.89, 0.996) | 0·17 |
| **RCRI only** | 0.34 | 0·78  (0·45, 0·94) | 0·48  (0·39, 0·57) | 0.97  (0.89, 0.99) | 0·18 |
| **CAD-RADS only (expert)** | 0.15 | 1·0  (0·70, 1·00) | 0·15  (0·10, 0·23) | 1.0  (0.82, 1.00) | 0·15 |
| **CAD-RADS only (DL)** | 0.43 | 0·89  (0·57, 0·98) | 0·30  (0·23, 0·39) | 0.97  (0.86, 0.995) | 0·16 |
| **RCRI + patient data** | 0.54 | 0·56  (0·27, 0·81) | 0·71  (0·63, 0·79) | 0.96  (0.89, 0.98) | 0·21 |
| **CAD-RADS (expert) + patient data** | 0.01 | 0·56  (0·27, 0·81) | 0·78  (0·68, 0·83) | 0.96  (0.90, 0.98) | 0·24 |
| **CAD-RADS**  **(DL) + patient data** | 0.11 | 0·89  (0·57, 0·98) | 0·45  (0·37, 0·54) | 0.98  (0.91, 0.997) | 0·20 |
| **Multimodal** | | | | | |
| **Tabular data [CAD-RADS (DL) + patient data)]** | 0.35 | 0·78  (0·45, 0·94) | 0·51  (0·38, 0·56) | 0.97  (0.89, 0.99) | 0·18 |
| **LV + Heart + Tabular** | 0.06 | 0·89  (0·57, 0·98) | 0·53  (0·45, 0·62) | 0.99  (0.92, 0.997) | 0·18 |
| **LV + Aorta + Tabular** | 0.13 | 0·89  (0·57, 0·98) | 0·58  (0·49, 0·66) | 0.99  (0.93, 0.998) | 0·23 |
| **LV + Heart + Aorta + Tabular** | 0.70 | 0·83  (0·55, 0·94) | 0·79  (0·69, 0·85) | 0.98  (0.94, 0.995) | 0·43 |

LV, left ventricle; CI, confidence interval; CAD-RADS, coronary artery disease - reporting and data system

**Table S4. Performance of different models in predicting 30-day type 2 myocardial infarction. The numbers in parentheses indicate the 95% confidence intervals (CI).**

| **Input** | **AUC-ROC ↑** | **Sensitivity*↑** | **Specificity*↑** | **NPV*↑** | **F1-score*↑** |
| --- | --- | --- | --- | --- | --- |
| **CAD-RADS versus RCRI and patient data (demographics, history of vascular disease, and vascular risk factors)** | | | | | |
| **Patient data** | 0.58  (0.37, 0.79) | 0.50  (0.22, 0.78) | 0.71  (0.62, 0.78) | 0.96  (0.89, 0.98) | 0.17 |
| **RCRI only** | 0.55  (0.36, 0.74) | 0.78  (0.45, 0.94) | 0.50  (0.42, 0.59) | 0.97  (0.89, 0.99) | 0.13 |
| **CAD-RADS only (expert)** | 0.56  (0.35, 0.78) | 0.50  (0.22, 0.78) | 0.69  (0.60, 0.77) | 0.95  (0.89, 0.98) | 0.16 |
| **CAD-RADS only (automated)** | 0.57  (0.36, 0.79) | 0.38  (0.14, 0.69) | 0.77  (0.68, 0.83) | 0.95  (0.89, 0.98) | 0.15 |
| **RCRI + patient data** | 0.59  (0.37, 0.81) | 0.88  (0.53, 0.98) | 0.41  (0.32, 0.50) | 0.98  (0.90, 1.00) | 0.16 |
| **CAD-RADS (expert) + patient data** | 0.60  (0.42, 0.79) | 0.38  (0.14, 0.69) | 0.88  (0.80, 0.93) | 0.95  (0.90, 0.98) | 0.23 |
| **CAD-RADS**  **(automated) + patient data** | 0.61  (0.47, 0.74) | 1.00  (0.68, 1.00) | 0.42  (0.33, 0.51) | 1.00  (0.93, 1.00) | 0.19 |
| **Multimodal** | | | | | |
| **Tabular data [CAD-RADS (DL) + patient data]** | 0.63  (0.44, 0.81) | 0.88  (0.53, 0.98) | 0.45  (0.36, 0.54) | 0.98  (0.90, 1.00) | 0.17 |
| **LV + Heart + Tabular** | 0.71  (0.54, 0.89) | 0.63  (0.31, 0.86) | 0.82  (0.74, 0.88) | 0.97  (0.92, 0.99) | 0.29 |
| **LV + Aorta + Tabular** | 0.73  (0.52, 0.96) | 0.75  (0.41, 0.93) | 0.77  (0.68, 0.83) | 0.99  (0.93, 0.99) | 0.29 |
| **LV + Heart + Aorta + Tabular** | 0.75  (0.62, 0.89) | 0.49  (0.40, 0.58) | 1.00  (0.68, 1.00) | 1.00  (0.94, 1.00) | 0.21 |

Table S4 summarizes the performance of the RCRI, CAD-RADS, and combined clinical-imaging models when type 2 MI alone is used as the outcome. AUC with 95% confidence intervals and sensitivity, specificity, negative predictive value and F1 score are provided. Discrimination for type 2 MI was further reduced and estimates were less precise, with wider CI, reflecting the smaller number and greater clinical heterogeneity of type 2 MI events.

For type 2 MI, the multimodal DL model did not significantly outperform either RCRI (delta-AUROC = 0.15; 95% CI: −0.05–0.35; p = 0.11) or CAD-RADS (delta-AUROC = 0.14; 95% CI: −0.13–0.44; p = 0.10). This likely reflects limited statistical power attributable to the smaller number of type 2 MI events.

**Table S5. Performance of different models in predicting 30-day composite outcome including heart failure. The numbers in parentheses indicate the 95% confidence intervals (CI).**

| **Input** | **AUC-ROC ↑** | **Sensitivity*↑** | **Specificity*↑** | **NPV*↑** | **F1-score*↑** |
| --- | --- | --- | --- | --- | --- |
| **CAD-RADS versus RCRI and patient data (demographics, history of vascular disease, and vascular risk factors)** | | | | | |
| **Patient data** | 0.63  (0.45, 0.82) | 0.78  (0.45, 0.94) | 0.52  (0.43, 0.61) | 0.97  (0.89, 0.99) | 0.19 |
| **RCRI only** | 0.60  (0.41, 0.78) | 0.78  (0.45, 0.94) | 0.50  (0.42, 0.59) | 0.97  (0.89, 0.99) | 0.19 |
| **CAD-RADS only (expert)** | 0.61  (0.45, 0.78) | 0.72  (0.63, 0.80) | 0.56  (0.27, 0.81) | 0.96  (0.89, 0.98) | 0.21 |
| **CAD-RADS only (automated)** | 0.62  (0.47, 0.77) | 0.89  (0.57, 0.98) | 0.34  (0.27, 0.43) | 0.98  (0.88, 1.00) | 0.17 |
| **RCRI + patient data** | 0.65  (0.48, 0.82) | 0.89  (0.57, 0.98) | 0.45  (0.37, 0.54) | 0.98  (0.90, 1.00) | 0.20 |
| **CAD-RADS (expert) + patient data** | 0.65  (0.47, 0.83) | 0.78  (0.45, 0.94) | 0.65  (0.56, 0.73) | 0.97  (0.91, 0.99) | 0.24 |
| **CAD-RADS**  **(automated) + patient data** | 0.66  (0.50, 0.81) | 0.89  (0.57, 0.98) | 0.46  (0.38, 0.55) | 0.98  (0.91, 1.00) | 0.20 |
| **Multimodal** | | | | | |
| **Tabular data [CAD-RADS (DL) + patient data]** | 0.67  (0.51, 0.84) | 0.67  (0.35, 0.88) | 0.74  (0.65, 0.81) | 0.97  (0.91, 0.99) | 0.26 |
| **LV + Heart + Tabular** | 0.70  (0.50, 0.89) | 0.67  (0.35, 0.88) | 0.76  (0.67, 0.82) | 0.97  (0.91, 0.99) | 0.27 |
| **LV + Aorta + Tabular** | 0.73  (0.59, 0.87) | 0.78  (0.45, 0.94) | 0.71  (0.63, 0.79) | 0.98  (0.92, 0.99) | 0.28 |
| **LV + Heart + Aorta + Tabular** | 0.80  (0.69, 0.91) | 1.00  (0.70,1.00) | 0.63  (0.54, 0.71) | 1.00  (0.95, 1.00) | 0.29 |

In Table S5, for each model, the mean AUC with 95% confidence intervals is shown, together with sensitivity, specificity, negative predictive value, and F1-score at the Youden index as thresholds. Compared with their performance for the primary MACE endpoint, discrimination for the broader 30-day composite outcome was slightly lower across all models.

For prediction of composite outcome including heart failure, the multimodal deep learning (DL) model demonstrated superior discrimination compared with RCRI (delta-AUROC = 0.20; 95% CI 0.02–0.40; p = 0.04). However, the improvement over CAD-RADS did not reach statistical significance (ΔAUROC = 0.19; 95% CI: −0.05–0.46; p = 0.12).

**Figure S1. The architecture of Perceiver encoder and decode**


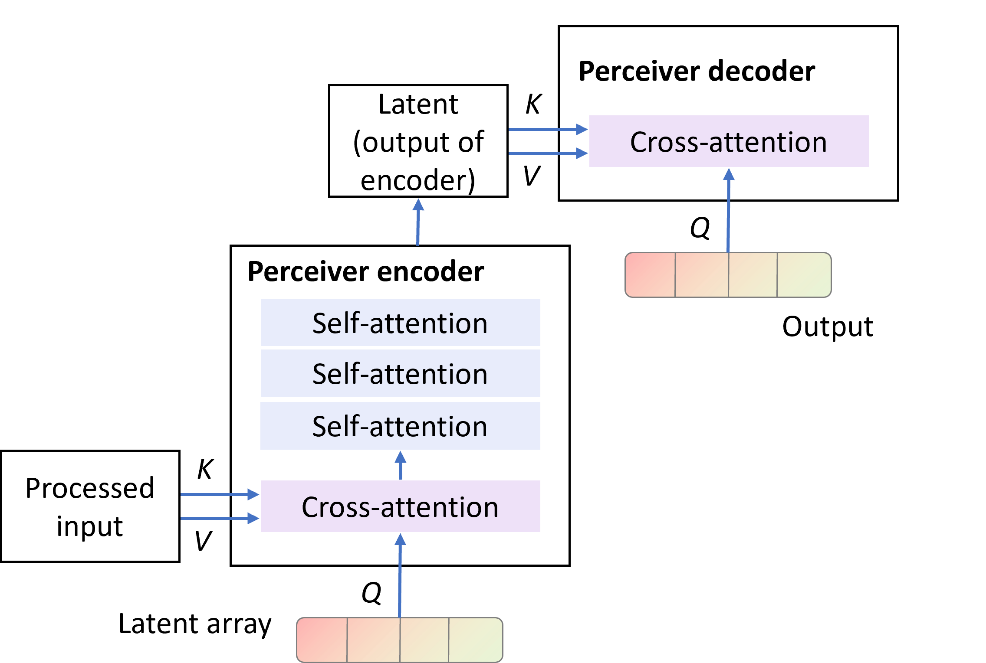


**Figure S2. Calibration plot**


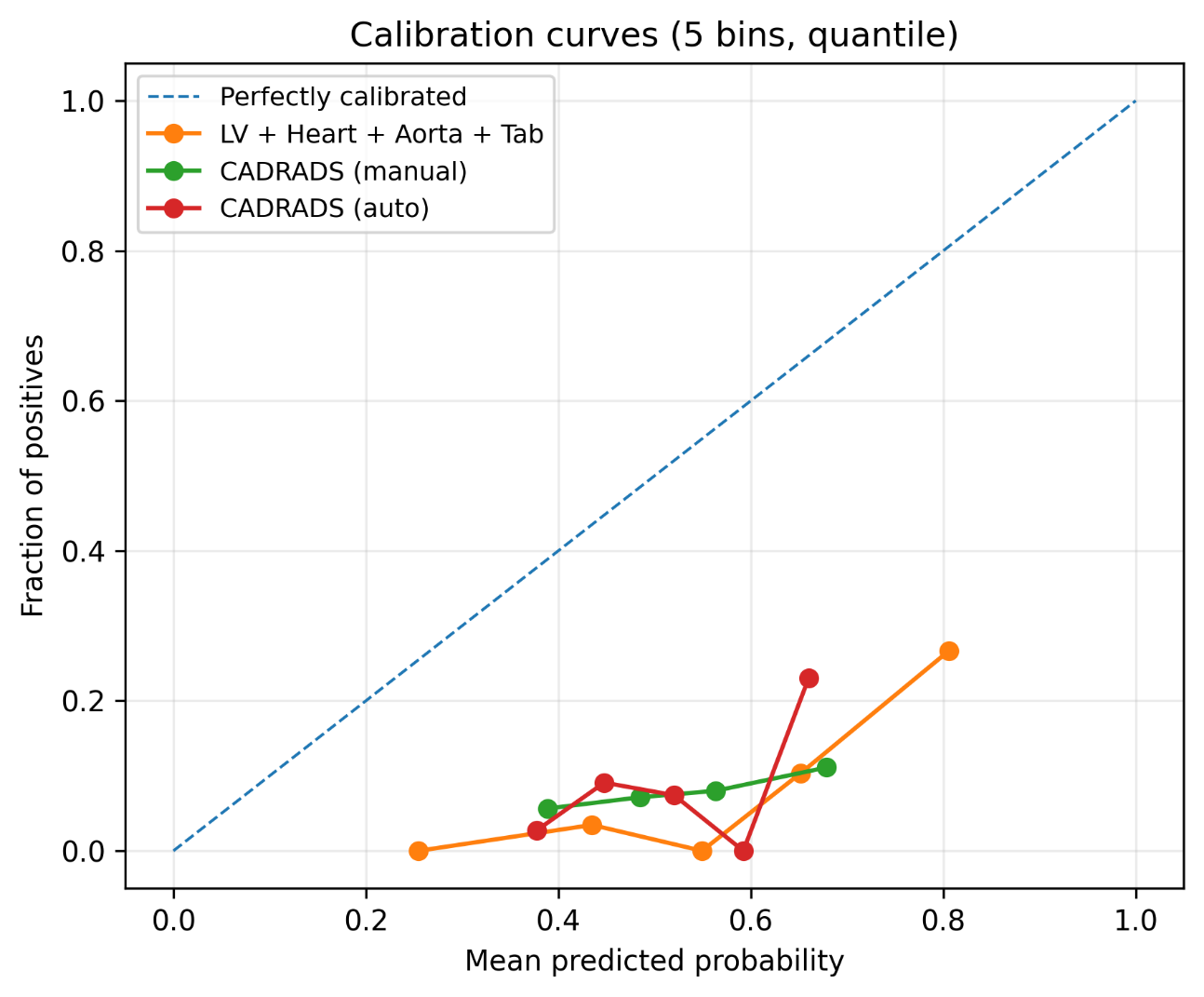


**Figure S3. Decision curve analysis**


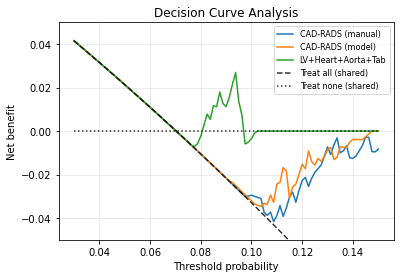

Supplement: ztag037_Supplementary_Data [file ztag037_supplementary_data.docx]
